# Understanding the defense mechanism of *Allium* plants through the onion isoallicin-omics study

**DOI:** 10.3389/fpls.2024.1488553

**Published:** 2024-12-11

**Authors:** Heejung Cho, Ji Yeon Park, Duck Hyun Kim, JiWon Han, Karthi Natesan, Min-Seon Choi, Seon-Kyeong Lee, Jiseon Kim, Kun Cho, Byoung Ohg Ahn

**Affiliations:** ^1^ Genomics Division, National Institute of Agricultural Sciences, Rural Development Administration, Jeonju, Republic of Korea; ^2^ Digital Omics Research Center, Korea Basic Science Institute (KBSI), Cheongju, Republic of Korea; ^3^ Allium Vegetable Research Center, National Institute of Horticultural and Herbal Science, Rural Development Administration, Muan, Republic of Korea; ^4^ Metabolic Engineering Division, National Institute of Agricultural Sciences, Rural Development Administration, Jeonju, Republic of Korea

**Keywords:** *Allium cepa*, onion, isoallicin, defense mechanism, DHW30006 onion, alliinase

## Abstract

Onion (*Allium cepa* L.) is an important seasoning vegetable worldwide. It belongs to the Allium genus, which produces distinctive flavor compounds, allicin/isoallicin. It has been known that allicin/isoallicin is produced upon cell damage by vacuole-localized alliinase releasing. Pungent isoallicin and lachrymaroty factor (LF) are unique features of onions. To understand the isoallicin system of onions, we identified and characterized the biosynthesis-related genes by displaying transcriptional profiles and analyzing the isoallicin contents of onion plants. The DHW30006 onion genome encoded 64 alliinase (ALL) and 29 LF synthase (LFS) proteins, which are the key enzymes for producing of isoallicin and LF. Interestingly, when we analyzed the N-terminal signal peptide sequences (SP) necessary for transport to the vacuole, we found that 14 ALLs contained the SP (SP-ALL) and 50 ALLs did not (non-SP-ALL). We hypothesized that non-SP-ALLs stayed in the cytosol of onion cells, reacted with isoalliin, and generated isoallicin without cell damage. Our transcriptome and LC-MS/MS analyses reveal that isoallicin levels vary significantly across onion tissues and growth stages, with substantial production occurring in intact cells through cytosolic alliinases and an increase through vacuolar alliinases upon tissue disruption. This novel observation suggests that the isoallicin system in onions functions as a dual-defense mechanism: cytosolic alliinases maintain a constant level of defense against biotic stress in undamaged tissues, while vacuolar alliinases enhance isoallicin production in response to tissue damage by herbivory and insects. Together, these coordinated mechanisms demonstrate an adaptable and dynamic defense strategy against biotic stresses in Allium plants.

## Introduction

The genus *Allium* is very large, comprising almost 1,000 species, including important cultivated vegetables, such as bulb onion (*A*. *cepa*), garlic (*Allium sativum*), bunching onion (*Allium fistulosum*), Chinese chive (*Allium tuberosum*), leek (*Allium porrum*), and rakkyo (*Allium chinense*) (Shigyo et al., 2018). Alliaceous plants produce various sulfur-containing secondary metabolites, which confer distinctive flavors and medicinal effects (Borlinghaus et al., 2014). The antimicrobial activity of Allium plants has been studied for a long time due to their important roles in human disease prevention, food preservation and crop protection (Cavallito et al., 1944; Small et al., 1947). The extracts of Allium plants showed strong antimicrobial effects on viruses (Weber et al., 1992; Ankri and Mirelman, 1999), bacteria (Cavallito et al., 1944; Small et al., 1947; Zohri et al., 1995; Ankri and Mirelman, 1999; Yu, 1999; Whitemore and Naidu, 2000; Benkeblia, 2004; Curtis et al., 2004; Corzo-Martínez et al., 2007), fungi (Yoshida et al., 1987; Zohri et al., 1995; Ankri and Mirelman, 1999; Phay et al., 1999; Benkeblia, 2004; Curtis et al., 2004; Kim et al., 2004; Shadkchan et al., 2004; Davis, 2005; Gruhlke et al., 2010; Bagiu et al., 2012), parasites (Ankri and Mirelman, 1999), and nematodes (Nath et al., 1982; Tada et al., 1988; Gupta and Sharmaj, 1993; Nidiry et al., 1994; D’Addabbo et al., 2023). Also, it had repellent activities against insects, herbivores, and even birds (Mason and Linz, 1997; Jarial, 2001; Hile et al., 2004; Hosamani et al., 2007). The major compounds in the extracts are allicin/isoallicin, which are bioactive sulfur species mediated by redox-dependent reactions (Yoshimoto and Saito, 2019).

Alliaceous plants accumulate inactive precursor S-alk(en)ylcysteine S-oxides (ACSOs) such as alliin, isoalliin, methiin, cycloalliin, etc (Yamazaki et al., 2010; Yoshimoto and Saito, 2019). When tissue damage occurs, vacuole-stored alliinase is released and reacts with ACSOs (Lancaster and Collin, 1981; Ellmore and Feldberg, 1994; Manabe et al., 1998; Yamazaki et al., 2002). Resulting from chemical reactions cascade, allicin/isoallicin are produced, and damaged tissues release these and derived sulfur compounds (Block, 2010; Nwachukwu and Slusarenko, 2014). Allicin (diallylthiosulfinate) derived from alliin of garlic is the best known thiosulfinate. Because no further energy consumption occurs for allicin production, it belongs to “phytoanticipin”, which the term is “…low molecular weight, antimicrobial compound present in plants before challenge by microorganisms, or produced after infection solely from pre-existing constituents.” (Vanetten et al., 1994; Block, 2010). In onion, the major thiosulfinate is isoallicin derived from isoalliin (Block, 2010; Nwachukwu and Slusarenko, 2014; Böttcher et al., 2017; Saviano et al., 2019). Isoalliin is hydrolyzed by alliinase, and the intermediate 1-propenylsulfenic acid (PSA) proceeds in one of two reactions: i) spontaneous condensation of two PSA molecules and isoallicin (di-1-propenyl thiosulfinate) production, or ii) biosynthesis of lachrymatory factor (LF, propanethiol S-oxide) by lachrymatory factor synthase (LFS) (Imai et al., 2002; Masamura et al., 2012). The volatile compound LF is a distinctive feature of onions, deterring herbivores by tear-inducing (Block, 2010; Nwachukwu and Slusarenko, 2014). Regarding antimicrobial properties, allicin/isoallicin and derived sulfur compounds of *Allium* plants would be produced primarily to protect the plants from environmental and biotic stresses.

To understand the isoallicin system of onions, we investigated the allicin/isoallicin metabolism-related genes of DHW30006 onion genome and performed transcriptional analysis according to onion tissues during bulb growing. Along with transcriptome profiles, the isoallicin contents were measured and presented to show correlation between transcriptome and isoallicin metabolome. Until now, it was known that allicin/isoallicin are produced upon tissue damage (Lancaster and Collin, 1981; Ellmore and Feldberg, 1994; Manabe et al., 1998; Yamazaki et al., 2002). However, through this Isoallicin-omics study, we showed that onion plants produced isoallicin under normal conditions, regardless of cell damage. It is presumed to be a protective mechanism of onion plants against biotic stresses known as a phytoanticipin.

## Results

### Isoalliin biosynthetic pathway-related genes

**Figure 1:**
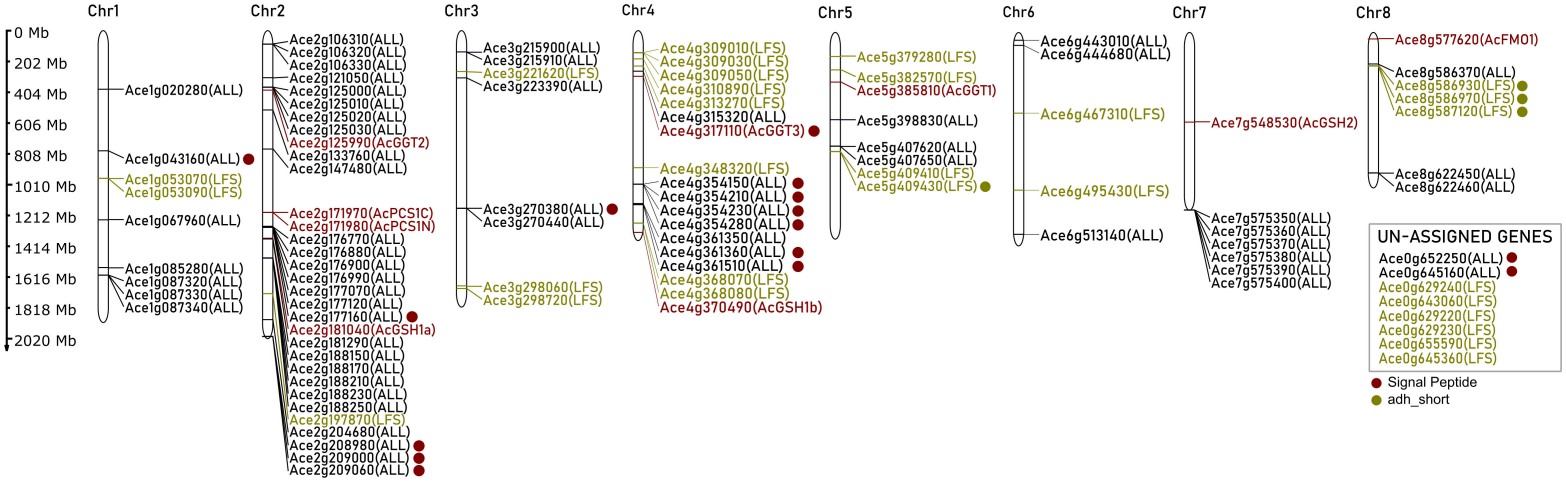
Chromosomal distribution of isoallicin biosynthetic pathway genes of DHW30006 onion genome. Dots indicate the signal peptide sequences (red) and the adh_short motif (dark yellow).

**Figure 2:**
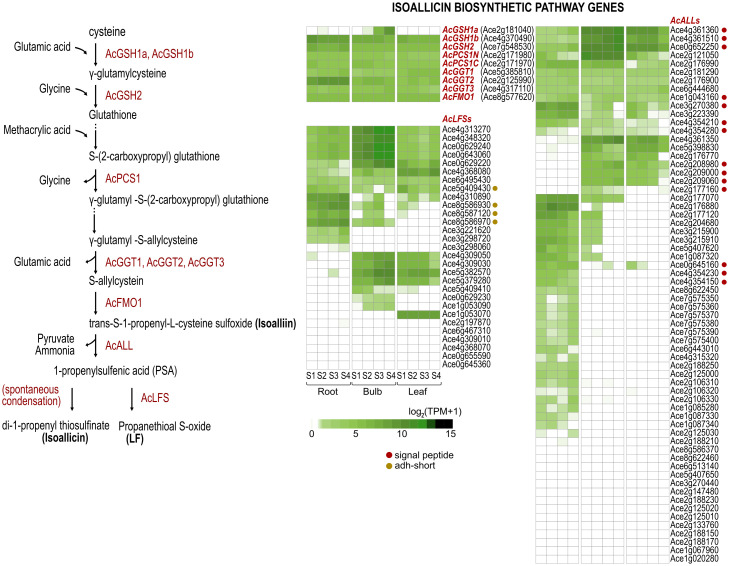
A simplified representation of the isoallicin biosynthetic pathway of onion and transcription profiles of the corresponding enzyme-encoding genes according to tissues and bulb growing stages (S1, S2, S3 and S4) of DHW30006 onion plants. The enzymes of each catalytic step are shown in red. Dots indicate the signal peptide (red) and the adh_short motif (dark yellow).

Sulfur-derived allicin/isoallicin are distinctive flavor compounds characteristic of the *Allium* genus. The biosynthetic pathway of isoalliin, the isoallicin precursor in onions, is not clear. Therefore, we identified candidate proteins of DHW30006 onion genome based on the proposed pathway proteins of garlic (Sun et al., 2020) through sequence homology searches. The DHW30006 onion genome contained two γ-glutamylcysteine synthetase orthologs (AcGSH1a and AcGSH1b), one glutathione synthetase (AcGSH2), a bicistronic phytochelatin synthase (AcPCS1 composed of AcPCS1N and AcPCS1C), three γ-glutamyl transpeptidase orthologs (AcGGT1, AcGGT2 and AcGGT3), and one allyl-cysteine S-oxygenase (AcFMO1) (**Figures 1**, **2**; **Supplementary Table S1**). In onion, phytochelatine synthase was bicistronic due to a single nucleotide variant (SNV) changes cytosine (C) in garlic to thymine (T), leading to a premature TAA stop codon (*AcPCS1N*), and then followed by a methionine start codon (*AcPCS1C*) (**Supplementary Figure S1**). This polycistronic configuration, which multiple proteins are encoded from a single mRNA transcript, is notable, as it is observed in virus, prokaryotes, plants and vertebrates (Hunt and Maiti, 2001; Karginov et al., 2017), and it suggests a unique adaptation in onion for this gene. Regarding GGT orthologs, based on a previous study in which the AsGGT2 protein was located in the vacuole of garlic (Yoshimoto et al., 2015), we screened the N-terminal signal peptide sequences (SP) (Rapoport, 2007) of isoalliin biosynthetic pathway proteins using SignalP5.0 (Almagro Armenteros et al., 2019) and found that AcGGT3 contained the SP, which indicated that AcGGT3 would function in the vacuole, while AcGGT1 and AcGGT2 would function in the cytosol of the onion cell. The nine isoalliin biosynthesis genes were dispersed across the chromosomes (**Figure 1**). Even the same functional orthologs (AcGSH1a and AcGSH1b, or AcGGT1, AcGGT2 and AcGGT3) were individually distributed across chromosomes.

### Alliinase and LF synthase gene family

**Figure 3:**
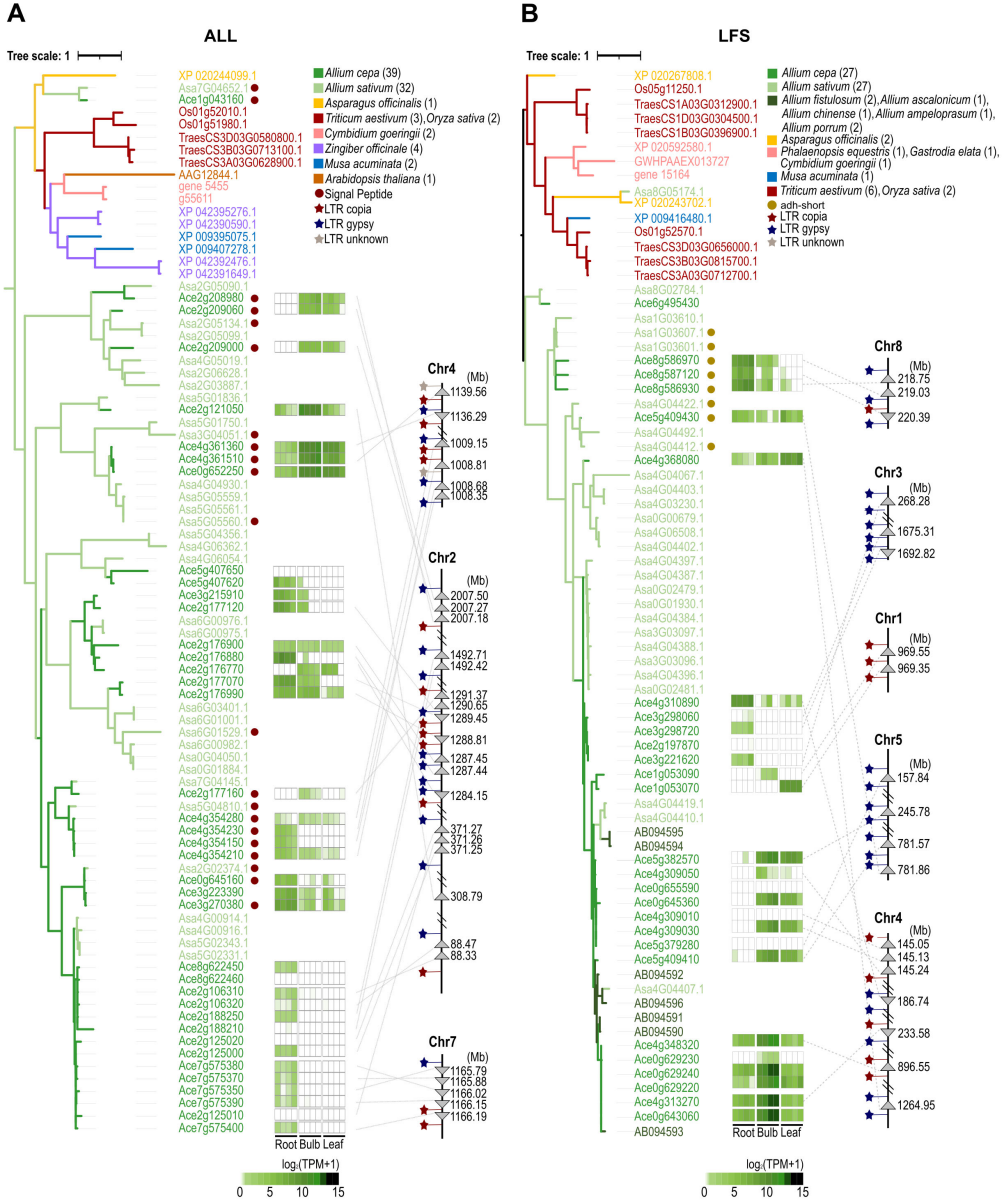
Integration of the genetic map, gene expression pattern and phylogenetic relationships of the onion ALL **(A)** and LFS **(B)** gene families. The phylogenetic tree was analyzed using the analogous protein sequences of each species, and the number of proteins is given in parentheses.

Pungent-flavored isoallicin and tear-inducing LF are unique features of onions. These compounds are produced by isoalliin hydrolysis via the action of alliinase (ALL) and the subsequent enzyme LF synthase (LFS), respectively (**Figure 2**). Motif analysis based on well-characterized ALL and LFS sequences identified 64 ALLs and 29 LFSs in onions (**Supplementary Tables S2**, **S3**). Non-*Allium* plants also contained ALL and LFS analogs, which might seem to participate in other sulfur metabolism pathways. To assess the phylogenetic relationships of ALL analogs, 39 onion ALLs, 32 garlic ALLs, and 15 alliinase orthologs of other plant species, including *A*. *officinalis*, *Cymbidium goeringii*, *Musa acuminata*, *Zingiber officinale*, *Oryza sativa*, *Triticum aestivum* and *Arabidopsis thaliana* were analyzed (**Figure 3A**; **Supplementary Tables S4**, **S5**). One onion ALL (Ace1g043160) and one garlic ALL (Asa7G04652.1) were clustered with non-*Allium* plant ALL analogs due to their high protein sequence identity; these genes were considered ancestral genes and played a role in common sulfur metabolism. All remaining onion and garlic ALLs were grouped either together with onion and garlic sequences or individually by species, which showed that *ALL* genes were amplified both before and after the differentiation of onion and garlic.

This amplification was also shown in the phylogenetic tree of LFS analogs of alliaceous plants and other monocotyledons, including 27 onion LFSs, 27 garlic, 2 A*. fistulosum* (bunching onion), one *A. chinense* (rakkyo), one *A. ascalonicum* (shallot), 2 A*. porrum* (leek), one *A. ampeloprasum* (elephant garlic), and 14 LFS analogs of other monocotyledons, including *A. officinalis*, *C. goeringii*, *G*. *elata*, *P*. *equestris*, *M*. *acuminata*, *O*. *sativa* and *T*. *aestivum* (**Figure 3B**; **Supplementary Tables S6**, **S7**). In the tree, one onion LFS (Ace6g495430) and two garlic LFSs (Asa8G05174.1 and Asa8G02784.1) were located close to other monocotyledon LFS analogs, which were expected to function in common sulfur metabolism rather than in LF synthesis. As observed in the ALL tree, the major onion and garlic LFSs were clustered either together or separately by Allium species, indicating that they expanded both before and after speciation. The gene expansion of the ALL and LFS families was clearly demonstrated by integrating the genetic map, gene expression patterns and phylogenetic tree of the onion ALL and LFS families (**Figure 3**). The chromosomal colocalized groups were also clustered together in the phylogenetic trees and showed similar expression patterns across different tissues. In the genetic map, a number of Gypsy/Copia-type LTRs were found adjacent to the clustered *ALL* and *LFS* genes (**Supplementary Tables S8**, **S9**). This indicates that onion *ALL* and *LFS* gene amplification has been mediated by these LTRs.

Based on a report about the vacuole localization of alliinase (Lancaster and Collin, 1981), we analyzed the N-terminal signal peptide sequences (SPs) of onion ALLs, which are essential for extracellular protein secretion or intracellular organelle protein transportation (Rapoport, 2007). Sixty-four onion ALLs were determined to be 14 SP-ALLs and 50 non-SP-ALLs (**Supplementary Table S2**). In garlic, sixty-one ALLs were divided into 13 SP-ALLs and 48 non-SP-ALLs (**Supplementary Table S4**). SP-ALLs are expected to be transported to vacuoles and perform their functions upon tissue damage. Non-SP-ALLs comprised 50 in onion and 48 in garlic, which accounted for approximately 78% of the total ALLs in both onion and garlic. They were thought to be involved in allicin/isoallicin production in the cytosol without cell disruption. Based on this, we hypothesized that non-SP-ALLs might produce allicin/isoallicin in the cytosol of undamaged cells and affect the surrounding environment through antimicrobial activity in ordinary time. We subdivided onion ALLs into vacuole alliinase (SP-ALL) and cytosol alliinase (non-SP-ALL) based on their functional locations.

Among the onion LFS proteins, four LFSs (encoded by Ace5g409430, Ace8g586930, Ace8g586970 and Ace8g587120) had an additional alcohol dehydrogenase short-chain (adh_short) motif (**Supplementary Table S3**). We could not predict the function of these proteins, but three of them are strongly expressed in the roots. This type of LFS was also found in garlic with four and clustered with onion LFSs in the phylogenetic tree (**Figure 3B**; **Supplementary Table S6**).

### Isoallicin metabolism of onion plants

**Figure 4:**
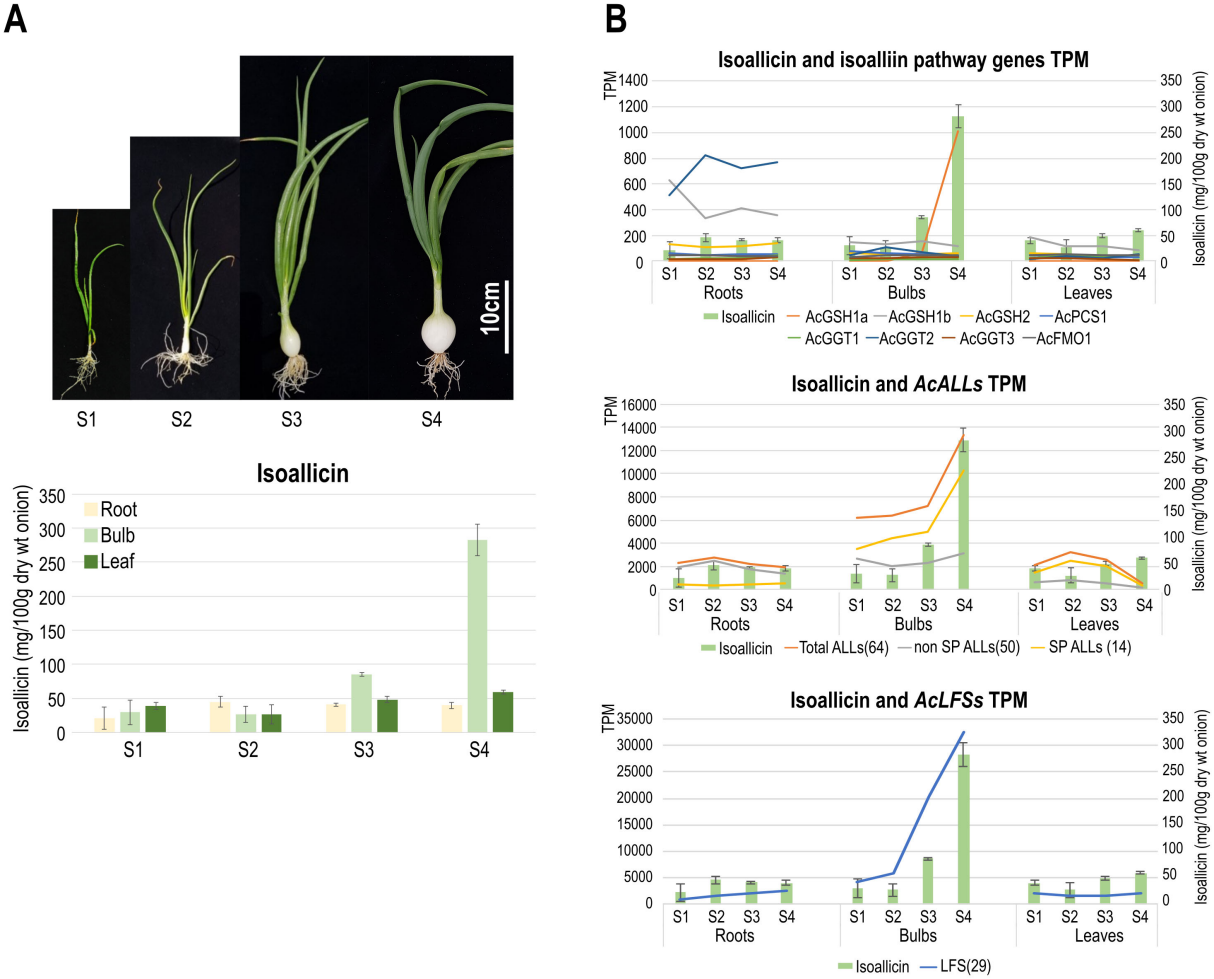
LC-MS/MS analysis of isoallicin in DHW30006 onion plants. **(A)** Isoallicin contents of field-grown onion plants according to tissues during bulb growing stages (S1, S2, S3 and S4). **(B)** Correlation of isoallicin contents and the expression (TPM) of putative isoalliin biosynthetic pathway (upper), *ALL* (middle) and *LFS* (lower) genes during bulb growing. S1 to S4 onion samples were extracted from lyophilized tissues by three replicates.

To investigate the relationship between gene expression and isoallicin production, we measured isoallicin content in onion plants using LC-MS/MS, along with transcriptional profiles of different tissues during the bulb growth stages (S1-S4) (**Figure 4**; **Supplementary Tables S10**–**S14**). In the isoallicin extraction process, we incubated homogenates for 30 minutes at room temperature (RT), which allowed the release of vacuole-localized alliinases to react with isoalliin and produce isoallicin. The results revealed significant variation in isoallicin levels across onion tissues and growth stages (**Figure 4A**), with the highest levels observed in S4 bulbs, showing a marked increase during bulb expansion (S3-S4).

We studied isoallicin contents together with transcriptional profiles according to the following functional stages: i) isoalliin biosynthesis pathway genes, ii) *ALL* genes, and iii) *LFS* genes (**Figure 4B**). Most isoalliin biosynthesis genes (*AcGSH2*, *AcPCS1*, *AcGGT1*, *AcGGT3* and *AcFMO1*) were expressed at basal levels in all tested tissues (**Figure 4B**_upper). The *AcGSH1a* gene was only expressed in expanding bulbs at very high level, which exhibited the highest isoallicin content. Therefore, AcGSH1a is considered to be an enzyme specialized for isoallicin production in growing onion bulbs. On the other hand, the *AcGSH1b* and *AcGGT2* genes showed significantly higher expression in all stages of roots compared to bulbs and leaves. This was thought to be related to the root’s role in absorbing organic sulfur compounds as well as isoallicin synthesis.

ALL and LFS, the key enzymes responsible for the production of isoallicin and LF, are multigene families. The transcription profiles of *ALL* and *LFS* genes were represented by the sum of transcripts per million (TPM) values for each gene of each family (**Figure 4B**). The expression of these gene families drastically increased as the bulb grew, accompanied by increasing isoallicin contents. Regarding *ALL* family genes, it was presented in detail by dividing into *SP-* and *non-SP-ALL* groups, which represented the effects of vacuolar and cytosolic alliinases, respectively. Each type of *ALL* was expressed differently according to tissues and growth stages (**Figure 4B**_middle). In the onion roots, the isoallicin content followed the *non-SP-ALL* expression values at all growth stages. *SP-ALL* genes were expressed, but *non-SP-ALLs* were predominant. This means that onion roots produce isoallicin mainly by cytosolic alliinase in ordinary time during plant growing and prepare for cell disruptions by maintaining the basal level of vacuolar alliinase expression. In onion bulbs, the expression of both types of *ALLs* was very active; the *non-SP-ALL* transcripts were present at similar levels to that of roots, and the *SP-ALL* transcripts were increased coupled with isoallicin content during bulb expansion. In onion leaves, *Non-SP-ALL* genes were barely expressed at all stages, whereas *SP-ALL* genes were predominantly expressed. The S4 leaves showed minimal gene expression, yet they had a notable presence of isoallicin, thought to have been transported from onion bulbs.

**Figure 5:**
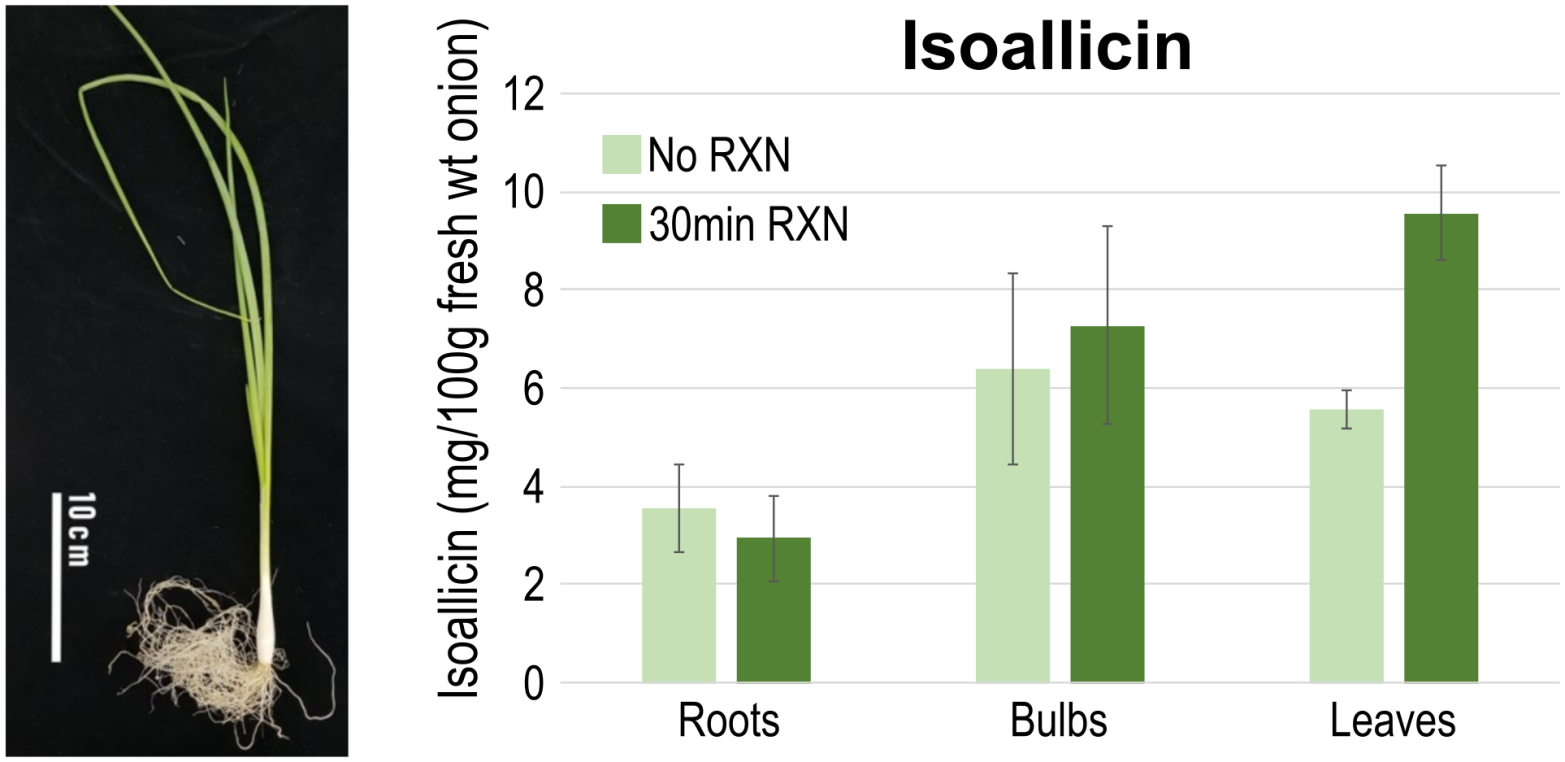
Isoallicin contents of onion tissues according to the extraction conditions. (No RXN) Direct extraction of onion tissues for vacuolar alliinase no-reaction and (30min RXN) 30 min incubation for vacuolar alliinase reaction. DHW30006 onion plants were grown in greenhouse for three months and samples were extracted from fresh tissues by four replicates.

With the presence and expression of non-SP-ALL type genes, isoallicin was expected to be present in onion tissues prior to cell disruption. To verify the functional activity of non-SP-ALLs (cytosolic alliinases), we quantified pre-existing isoallicin by directly extracting onion tissues under conditions where no enzyme reaction was allowed (No RXN), thereby excluding the effects of SP-ALLs (vacuolar alliinases) (**Figure 5**; **Supplementary Tables S15**, **S16**). The results showed that isoallicin was detected in all tested tissues under the No RXN condition, indicating that the cytosolic alliinases contribute significantly to isoallicin production in undamaged onion plants. Notably, onion roots, where non-SP-ALLs (cytosolic alliinases) were predominantly expressed, contained a significant amount of isoallicin. In the tissues, isoallicin levels were either similar or slightly higher in the No RXN condition compared to the 30-minute RXN. In contrast, bulbs and leaves showed higher isoallicin levels in the 30-minute RXN compared to No RXN, which reflects the activity of vacuolar alliinases in these tissues. This was corroborated by the transcriptome analysis, which indicated higher expression levels of SP-ALL genes than non-SP-ALLs in bulbs and leaves. The onion samples used in **Figure 5**, grown in a greenhouse for three months, exhibited a physiological state similar to the S1 stage onions in **Figure 4**, as indicated by tissue-specific isoallicin levels measured after a 30-minute reaction (i.e., isoallicin levels: roots < bulbs < leaves).

In summary, onion roots produce and contain isoallicin through the action of cytosolic alliinases, even before any tissue damage occurs. While bulbs and leaves also contain significant levels of isoallicin due to the cytosolic alliinases, they produce more isoallicin in response to damage by the vacuolar alliinases. This is considered a strategy to further protect against damage caused by herbivores. This dual-system strategy likely serves to enhance the plant’s defense mechanisms against attacks from microorganisms to herbivores.

## Discussion

In this study, we characterized the isoallicin system of onion plants by integrating genome, transcriptome and metabolome analyses. Two distinctive onion compounds, isoallicin and tear-inducing LF, are synthesized by *AcALL* and *AcLFS* gene families. Phylogenetic analyses combined with genomic locations, long terminal repeat (LTR) elements, and expression patterns highlighted the expansion of AcALLs and AcLFSs, which was especially prominent during onion bulb development.

Through transcriptome and LC-MS/MS analyses, we observed that isoallicin levels varied significantly across tissues and developmental stages in DHW30006 onion plants, suggesting that isoallicin biosynthesis is tightly regulated during growth. Specifically, expanding bulbs (S3-S4) increased the expression of *AcGSH1a*, *AcALLs*, and *AcLFSs* genes, along with a corresponding increase in isoallicin production. The high capacity for isoallicin production in onion bulbs appears to be an evolutionary adaptation that supports long-term storage and contributes to the preservation of the species. Ludlow et al. (2021) found that prolonged storage of garlic results in decreased alliinase activity and increased spoilage of garlic cloves. Janská et al. (2021) demonstrated that increased concentrations of alliinase enhanced antibacterial effects, highlighting the enzyme’s importance for antimicrobial protection. Like this, high accumulation of isoallicin production capacity in onion bulbs is essential for preserving them during dormancy, helping maintain viability for reproductive growth in the following year.

By varying the extraction conditions, we also demonstrated that isoallicin was produced regardless of cell disruption with two types of alliinases (cytosolic and vacuolar) and those exhibited distinct activities across different tissues. This finding reveals that onion plants can produce isoallicin during growth, whether injured or not, to protect themselves from biotic stresses. Previously, allicin production was thought to occur only upon cell damage, when alliinase is released from vacuoles (Block, 2010) and it was supported by the location of alliinase in the vacuole (Lancaster and Collin, 1981). However, our results suggest that onions have alliinases located in both the vacuole and the cytosol, allowing for continuous isoallicin production even without tissue injury. These enzymes work in tandem across different tissues to support isoallicin production continuously.

Our findings indicate that cytosolic and vacuolar alliinases fulfill different defensive roles based on tissue type and growth stage. Cytosolic alliinases, which exhibit higher activity in roots, are likely crucial for root defense against soil microbes. Onion roots, constantly interacting with rhizosphere microbes, produced substantial isoallicin even without cell disruption, suggesting a steady antimicrobial defense in healthy roots. In contrast, vacuolar alliinases showed higher activity in bulbs and leaves, where they may respond to tissue damage caused by herbivores or other threats. Although cytosolic alliinase levels are low in leaves, extracted samples contained significant amounts of isoallicin, likely transported from bulbs, which are primary isoallicin producers. Since allicin/isoallicin is produced in the bundle sheath of vascular tissue (Manabe et al., 1998; Yamazaki et al., 2002), it can be easily transported from bulbs to leaves through the vascular tissue. This tissue-specific system for isoallicin production and transfer appears to be an optimal defense mechanism, allowing bulbs to specialize in isoallicin production while leaves prioritize photosynthesis for energy production.

The defensive properties of *Allium* plants have long been used in eco-friendly agriculture such as intercropping/mixed cropping, to inhibit microbial pathogens or repel herbivorous predators from target crops (Tada et al., 1988; Zeng, 2008). It has been proven that intercropping with *Allium* plants, such as onions, garlic, leeks and chives, decreases pests and diseases in crops like cucurbits, sweet peppers, carrots, sugar beets, potatoes, tomatoes, and wheat by reducing insect populations and controlling pathogens (Uvah and Coaker, 1984; Potts and Gunadi, 1991; Yu, 1999; Amarawardana et al., 2007; Zeng, 2008; Zhou et al., 2013; Abdel-Baset, 2020; Baudry et al., 2021). Studies show that intercropping Allium plants can help control pests and diseases through the release of allicin/isoallicin. For instance, undamaged chive plants reduce aphid visitation on sweet peppers similarly to damaged plants, suggesting allicin/isoallicin release occur even without plant injury (Amarawardana et al., 2007). In carrot-onion mixed cropping, carrot fly attacks were reduced compared to carrot monoculture (Uvah and Coaker, 1984). The reduced presence of larvae and pupae in soil near carrot roots suggests that onion root exudates may impact the fly population, possibly through bioactive sulfur compounds like isoallicin. In tomato-Chinese chive intercropping, the number of wilted tomato plants, caused by the soil-borne pathogen *R*. *solanacearum*, was reduced compared to tomato monocropping (Yu, 1999). The study also tested the antibacterial effect of Chinese chive root exudates, finding that *R*. *solanacearum* growth was reduced when the exudates were added to the culture medium compared to the untreated medium. Those studies indicate that *Allium* plants release allicin/isoallicin during plant growth, regardless of damage. Therefore, the allicin/isoallicin production in *Allium* plants is important ecologically for sustainable agriculture by helping control biotic stresses.

**Figure 6:**
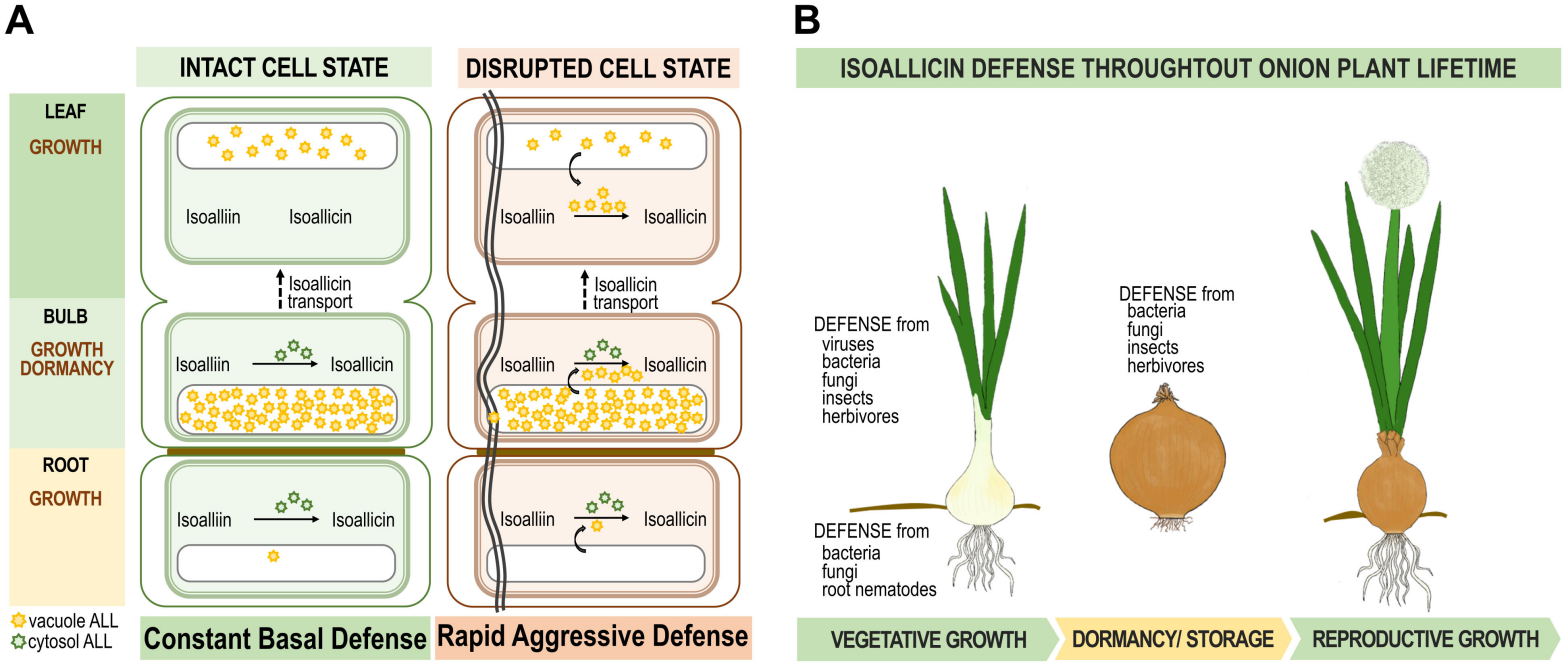
Proposal of isoallicin defense mechanism of onion plants. **(A)** Isoallicin production mechanism in intact and burst cell states according to onion tissues. **(B)** Isoallicin defence system throughout onion plant lifetime.

Based on these speculations, we developed a model of the isoallicin defense mechanism in onions plants with specific depictions for defense at the onion tissue level and the overall lifecycle of the plant (**Figure 6**). In **Figure 6A**, isoallicin production is depicted in two cellular states: intact and burst cells. In the intact cell state, the isoalliin in the cytosol is converted into isoallicin by cytosolic alliinase. In the burst cell state, vacuolar alliinase is released, leading to the rapid production of isoallicin, particularly in bulbs and leaves. This response mechanism indicates an immediate activation of the defense system upon tissue damage by insects and herbivores. **Figure 6B** provides an overview of the isoallicin defense system over the onion plant’s life cycle. It was demonstrated that the onion protects itself through the isoallicin mechanism during growth, dormancy, and reproductive growth, ensuring safe preservation of descendants.

Plants have developed diverse systems to defend against biotic stresses. These can be grouped into genetic, biochemical and physical defenses. Plants utilize these systems by appropriate combination according to their innate genetic system and environmental conditions. *Allium* plants are herbaceous so physically weak, but they equipped with the strong biochemical weapon allicin/isoallicin. Through the Isoallicin-omics study based on the DHW30006 onion genome, we enhanced our understanding of how onion plants defend themselves throughout their lifecycle against biotic stresses.

## Materials and methods

### Sample preparation, RNA sequencing and transcriptome analysis

For transcriptome analysis, DHW30006 onion plants (a short-day onion) were grown on the farm of the Allium Vegetable Research Center (belonging to the National Institute of Horticultural and Herbal Science) in Muan, South Korea (34°58´02.7’’N, 126°27´06.8’’E). For the preparation of RNA-seq for differential gene expression analysis, onion plants were sown on September 10, 2020. Sampling was harvested on March 5, 2021 (S1, 176 days after sowing), March 26, 2021 (S2, 197 days), April 16, 2021 (S3, 218 days), and May 7, 2021 (S4, 239 days), with samples collected from roots, bulbs, and leaves at each developmental stage, with three replicates. Harvested samples were frozen and ground in liquid nitrogen. Those stage samples were also harvested for measuring isoallicin contents with three replicates. Total RNAs were extracted using Trizol reagent (ambion, USA) according to the manufacturer’s instructions. To eliminate residual DNA fragments, the extracted RNA solution was treated with DNaseI (Qiagen, Germany) and further purified with an additional treatment of Trizol and Chloroform.

For RNA sequencing, the libraries were prepared according to the manufacturer’s instructions (Illumina Truseq stranded mRNA library prep kit). RNA sequencing is performed using an Illumina NovaSeq 6000 system following provided protocols for 2×151bp for differential expression analysis. RNAseq reads were preprocessed using Cutadapt v.2.8 (Martin, 2011) and mapped using the aligner STAR v2.7.1a (Dobin et al., 2013). Gene expression estimation was performed using RSEM v.1.3.1 (Li and Dewey, 2011). To normalize sequencing depth among samples, FPKM and TPM values were calculated. Based on the estimated read counts, differentially expressed genes (DEGs) were identified using the R package called TCC v.1.26.0 (Sun et al., 2013). Normalization factors were calculated using the iterative DESeq2 (Love et al., 2014)/edgeR (Robinson et al., 2010) method. The Q-value was calculated based on the p-value using the p.adjust function of the R package with default parameter settings. The DEGs were identified based on a q-value threshold less than 0.05 to correct errors caused by multiple testing (Benjamini and Hochberg, 1995). The RNA-seq datasets generated for this study can be found in the National Agricultural Biotechnology Information Center (NABIC, https://nabic.rda.go.kr/) under accession number NN-9065 ~ NN-9100.

### Gene family analysis

We identified the isoallicin biosynthesis-related genes using the *A*. *cepa* DHW30006 genome sequence and annotation information (available at the National Agricultural Biotechnology Information Center, https://nabic.rda.go.kr/genome/nolog/introductionPage.do?projectNo=141). The pathway of isoallicin biosynthesis in onion was not fully understood, therefore, it was based on the proposed pathway in garlic (Sun et al., 2020). The orthologs of the pathway were identified using BLASTP with the protein sequences of AsGSH1a (Asa0G01394.1), AsGSH1b (Asa6G04049.1), AsGSH1c (Asa6G00503.1), AsGSH1d (Asa6G00526.1), AsGSH2 (Asa3G01581.1), AsPCS1 (Asa0G04380.1), AsGGT1 (LC008010), AsGGT2 (LC008011), AsGGT3 (LC008012) and AsFMO1 (AB924383). To determine the gene family members of alliinase (ALL) and lachrymatory factor synthase (LFS), we analyzed the genome using HMMsearch and the Pfam database with default parameters to screen the candidate proteins (Johnson et al., 2010). The query motifs were PF04863 (EGF-like) and PF04864 (Alliinase C) for the ALL family, and PF10604 (Polyketide_cyc2) for the LFS family protein. Duplicated sequences were excluded by self-BLAST analysis. We analyzed functional proteins using the SignalP 5.0 server (https://services.healthtech.dtu.dk/service.php?SignalP-5.0) (Almagro Armenteros et al., 2019) to predict the presence of signal peptides in the isoallicin pathway genes.

For construction of the ALL phylogenetic tree, it was analyzed with 39 onion ALLs, 32 garlic ALLs, and 15 alliinase orthologues of other plants including *Asparagus officinalis*, *Cymbidium goeringii*, *Musa acuminata*, *Zingiber officinale*, *Oryza sativa*, *Triticum aestivum* and *Arabidopsis thaliana* (**Supplementary Table S5**). In the case of the LFS family, the analysis was conducted using 27 onion LFSs, 27 garlic (*A*. *sativum*), 2 A*. fistulosum* (Japanese bunching onion), one *A. chinense* (rakkyo), one *A. ascalonicum* (shallot), 2 A*. porrum* (leek), one *A. ampeloprasum* (elephant garlic) (Masamura et al., 2012), and 14 LFS analogs of other monocotyledons including *Asparagus officinalis*, *Cymbidium goeringii*, *Gastrodia elata*, *Phalaenopsis equestris*, *Musa acuminata*, *Oryza sativa* and *Triticum aestivum* (**Supplementary Table S7**). Protein sequences were retrieved from NCBI. Multiple sequence alignments (MSA) were performed using MAFFT v.7.464 and a phylogenetic tree was generated by IQ-TREE v1.6.8 (http://www.iqtree.org/) using a maximum likelihood method with 1,000 bootstrap iterations. Trees were visualized using the R Studio package (ver. 1.4.3) APE 5.6-2.

### Extraction of isoallicin

For LC-MS/MS analysis of isoallicin contents in onion tissues, sample solutions were extracted using a modified method of developed by Yamazaki et al. (2010). For onion bulb growing stages, lyophilized samples (400 mg) were ground with a mortar and pestle. Powder samples were homogenized with 10 mL of distilled water and sonicated for 45 sec (with intervals of 15 sec on/15 sec off) at 35% amplitude using an ultrasonicator (Sonics, USA) in an ice container. After incubating for 30 min at room temperature (RT, 20 - 25°C), the extracts were centrifuged at 13,000 rpm for 15 min at 4°C to separate cell debris. By adding 1.5 mL of Mixture A (a solution of anhydrous formic acid (1%; v/v): methanol (HPLC grade), 40:60) to 3.5 mL of supernatant, the extracts were diluted to 5 mL. After centrifugation at 8,000 rpm for 5 min at 4°C, the extracts were filtered using a 0.45 μm syringe filter. The filtered supernatants were eluted using a Supelclean™ LC-18 column (Sigma-Aldrich, Germany) for LC-MS/MS analysis. One mL of filtrate was added to a pre-activated and pre-chilled LC-18 cartridge and collected. Note that isoallicin is unstable at high temperatures, so the assay was carried out as quickly as possible and the extracts were stored at -70°C before LC-MS/MS analysis. To measure the contents of isoallicin according to vacuolar alliinase (SP-ALL) and cytosolic alliinase (non SP-ALL) reactions, we adopted two extraction conditions. To reduce errors, we reduced extraction steps by excluding freeze-drying of tissue samples and sonication of homogenates. Three-month-old onion plants grown in a greenhouse were harvested. Each tissue (roots, bulbs, leaves) was aliquoted to 400 mg of fresh weight with four replicates and then immediately frozen with liquid nitrogen in a 2 mL tube containing two 5 mm aluminum beads. Frozen sample tubes were set in pre-frozen 48-hole-blocks of grinder with liquid nitrogen and ground three times using the 2010 Geno/Grinder^®^ (SPEX SamplePrep, USA) at 900 rpm for 1 min. We prepared the onion extracts from roots, bulbs, and leaves by two different extraction conditions. For isoallicin contents produced by vacuolar alliinases after cell disruption (30min RXN), frozen ground samples were homogenized with 500 μL of DW and incubated for 30 min at RT to allow the vacuolar alliinases reaction. Homogenates were then mixed with 250 μL of a mixture containing 1% formic acid solution and MeOH (40:60) and extracted. To quantify isoallicin previously produced by cytosolic alliinases (No RXN), frozen ground samples were homogenized with 750 μL ice-cold mixture [500 μL DW and 250 μL of a mixture 1% formic acid solution and MeOH (40:60)]. The samples were then extracted immediately without a 30-min incubation at RT to prevent further reactions by vacuolar alliinases following cell disruption. Further extraction processes (centrifugation, filtration and LC-18 column elution) were the same as described above.

### LC-MS/MS analysis of isoallicin

LC-MS analysis was conducted using an Agilent 1200 liquid chromatography (HPLC) system (Agilent, USA) coupled with a 6460 triple quadrupole mass spectrometer (QQQ) (Agilent, USA). Chromatographic separation occurred at 40°C on a Poroshell 120 EC-C18 column (3.0 mm × 150 mm, 2.7 μm, Agilent, US). The chromatographic conditions were as follows: a flow rate of 0.3 mL/min, a sample injection volume of 5 μL, using mobile phase A (0.1% formic acid in water) and mobile phase B (0.1% formic acid in acetonitrile). The gradient elution program was set as follows: 0-3.0 min, 10% to 50% B; 3.0-7.0 min, 50% to 50% B; 7.0-7.5 min, 50% to 10% B; 7.5-15.0 min, 10% to 10% B. The samples were diluted two-fold, and the injection volume was fixed at 3μL for all the analyses. The mass spectrometer operated in electrospray ionization (ESI) positive mode utilizing single reaction monitoring (SRM) with a capillary voltage set at 3500 V. We used the reference standard with allicin (CAS No. 539-86-6) (Santa Cruz, USA). To determine whether allicin is a suitable standard for measuring isoallicin, we compared the peak patterns of allicin, garlic extract, and onion extract. The peak patterns were analyzed in the condition in positive ion mode ([M+H]+), targeting the m/z 163 > 73 transition (**Supplementary Figure 2**). Various collision energies in the product ion mode were compared to enhance the detection and quantification of fragments. The MS/MS spectra illustrating the product ions for allicin, garlic extract, and onion extract are depicted in **Supplementary Figure 2**. Using LC-MS/MS analysis, we confirmed that allicin, garlic extract, and onion extract exhibited similar peak patterns at 45.2 m/z and 73.1 m/z (or 74.2 m/z) when fragmented from 163 m/z. The isoallicin contents were measured at 73.1 m/z.

### Determination of linearity, LOD, and LOQ and data analysis

The determination of linearity, limit of detection (LOD), and limit of quantification (LOQ) was conducted. LOD and LOQ were established based on the calibration curve used to assess linearity. The calibration curve was measured thrice to derive the average slope (S) and standard deviation of the y-intercept (σ). LOD was calculated as 3 * σ/S, and LOQ was calculated as 3.3 * LOD. The linearity of the calibration curve was assessed using the coefficient of determination (r²). For allicin, linearity was evaluated within concentrations ranging from 0.0064 to 100 mg/L in the experiments. Confirmation of peak retention times in chromatograms was carried out following sample processing in accordance with predefined pre-treatment methods.

The Agilent MassHunter software was utilized to manage all data, and peak areas were integrated by the software, serving as the basis for quantification.

## Data Availability

The RNA-seq datasets generated for this study can be found in the National Agricultural Biotechnology Information Center (NABIC, https://nabic.rda.go.kr/) under accession number NN-9065 ~ NN-9100. Further inquiries can be directed to the corresponding author/s.
