# Supplementary material for: Understanding the defense mechanism of Allium plants through the onion isoallicin-omics study
Source: Front Plant Sci. 2024 Dec 11;15:1488553. doi: 10.3389/fpls.2024.1488553 (PMC11668612; doi:10.3389/fpls.2024.1488553)
Supplement: Supplementary file 1 [file DataSheet1.docx]

Supplementary Material

## Supplementary Figures


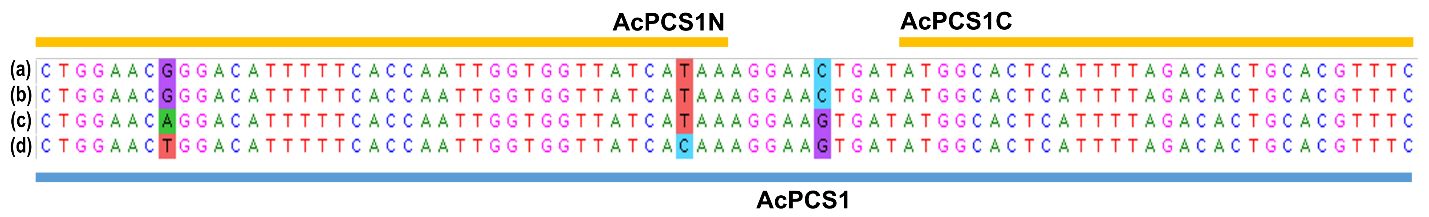


**Supplementary Figure 1.** Genome sequence alignment of intergenic region of AcPCS1N and AcPCS1C encoding genes of onions. (a) DHW30006 onion genome, (b) DHCU066619 onion genome, (c) red onion cultivar Eumjinara transcript, (d) garlic cultivar Ershuizao (*AsPCS1* gene). Thick yellow and blue line mean the protein products.


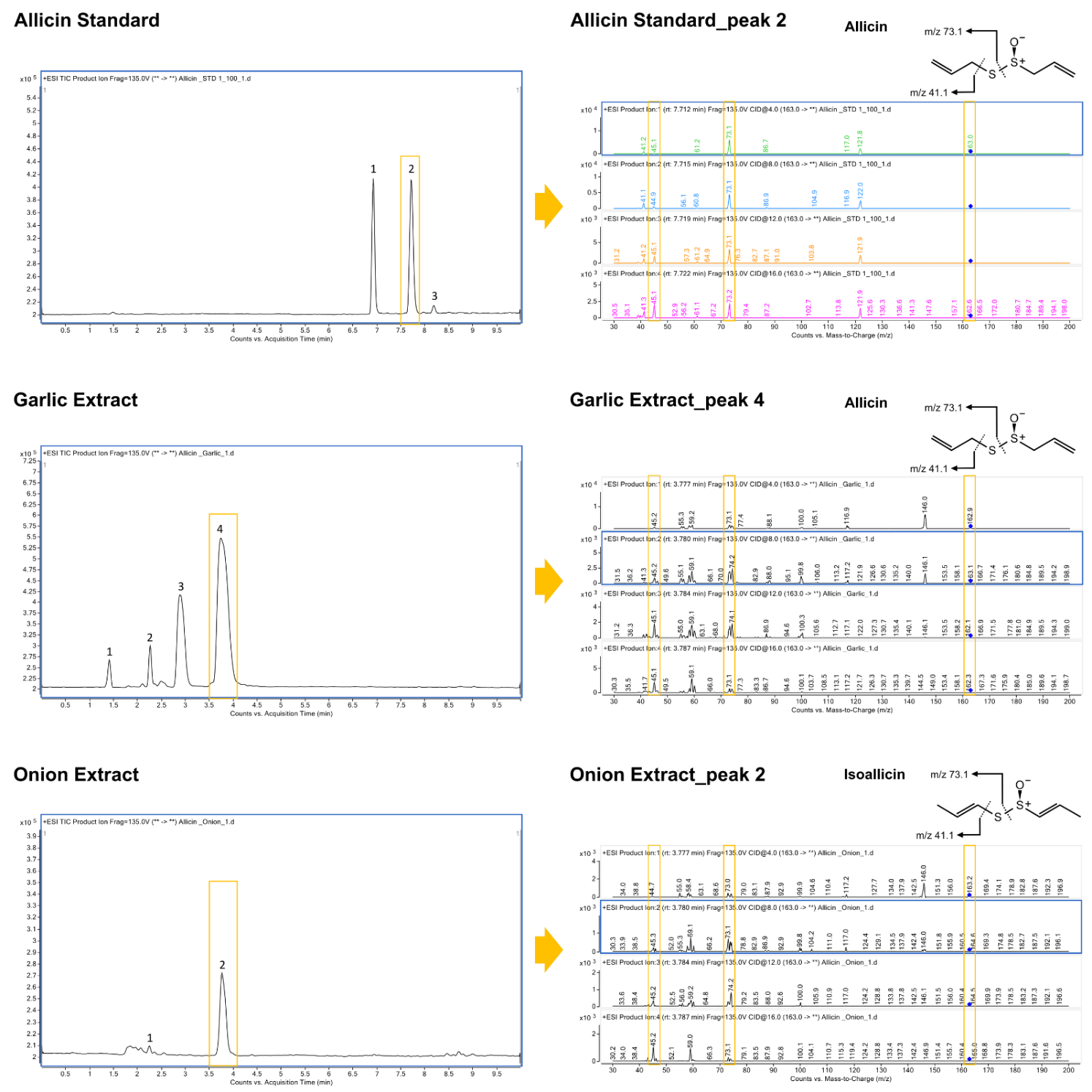


**Supplementary Figure 2.** Comparison of LC-MS/MS peak pattern of allicin standard, garlic extract and onion extract.
